# EML4-ALK fusion protein in Lung cancer cells enhances venous thrombogenicity through the pERK1/2-AP-1-tissue factor axis

**DOI:** 10.1007/s11239-023-02916-5

**Published:** 2023-11-08

**Authors:** Yanping Su, Jiawen Yi, Yuan Zhang, Dong Leng, Xiaoxi Huang, Xinyu Shi, Yuhui Zhang

**Affiliations:** 1 Department of Respiratory and Critical Care Medicine, Beijing Chao-Yang Hospital, Capital Medical University, Beijing, 100020 China; 2 Department of Respiratory and Critical Care Medicine, The First Affiliated Hospital of Zhengzhou University, Zhengzhou, 450052 China; 3 Clinical Laboratory, Beijing Chao-Yang Hospital, Capital Medical University, Beijing, 100020 China; 4 Basic Medical Research Center, Beijing Chao-Yang Hospital, Capital Medical University, Beijing, 100020 China

**Keywords:** EML4-ALK fusion protein, Tissue factor, Venous thromboembolism

## Abstract

**Background:**

Accumulating evidence links the echinoderm microtubule-associated protein-like 4 (EML4)-anaplastic lymphoma kinase (ALK) rearrangement to venous thromboembolism (VTE) in non-small cell lung cancer (NSCLC) patients. However, the corresponding mechanisms remain unclear.

**Method:**

High-throughput sequencing analysis of H3122 human ALK-positive NSCLC cells treated with ALK inhibitor/ dimethyl sulfoxide (DMSO) was performed to identify coagulation-associated differential genes between EML4-ALK fusion protein inhibited cells and control cells. Sequentially, we confirmed its expression in NSCLC patients’ tissues and in the plasma of a subcutaneous xenograft mouse model. An inferior vena cava (IVC) ligation model was used to assess clot formation potential. Additionally, pathways involved in tissue factor (TF) regulation were explored in ALK-positive cell lines H3122 and H2228. Statistical significance was determined by Student t-test and one-way ANOVA using SPSS.

**Results:**

Sequencing analysis identified a significant downregulation of TF after inhibiting EML4-ALK fusion protein activity in H3122 cells. In clinical NSCLC cases, TF expression was increased especially in ALK-positive NSCLC tissues. Meanwhile, H3122 and H2228 with high TF expression exhibited shorter plasma clotting time and higher TF activity versus ALK-negative H1299 and A549 in cell culture supernatant. Mice bearing H2228 tumor showed a higher concentration of tumor-derived TF and TF activity in plasma and the highest adjusted IVC clot weights. Limiting EML4-ALK protein phosphorylation downregulated extracellular regulated protein kinases 1/2 (ERK1/2)-activating the protein-1(AP-1) signaling pathway and thus attenuated TF expression.

**Conclusion:**

EML4-ALK fusion protein may enhance venous thrombogenicity by regulating coagulation factor TF expression. There was potential involvement of the pERK1/2-AP-1 pathway in this process.

**Supplementary Information:**

The online version contains supplementary material available at 10.1007/s11239-023-02916-5.

## Introduction

In recent years, accumulating studies have indicated a close link between genetic alterations and venous thromboembolism (VTE) occurrence in patients with malignant tumors [1–3]. Our team also explored the relationship between driver oncogene alterations and VTE event occurrence in non-small cell lung cancer (NSCLC) patients through a prospective cohort study. The results showed that the anaplastic lymphoma kinase (ALK) gene rearrangement in NSCLC conferred a significant increase in VTE risk [4, 5], which was consistent with previous retrospective studies. Sequentially, it was also verified again by professor Hanny et al. in a cohort study [6]. However, little is known about the specific mechanism of ALK rearrangement in NSCLC cells regulating VTE occurrence.

ALK rearrangement is the first somatic oncogene translocation discovered in lung cancer. In 2007, a Japanese team identified a fusion gene caused by inversion within chromosome 2p, comprising portions of the echinoderm microtubule-associated protein-like 4 (EML4) gene located in p21 and the ALK gene located in p23 in NSCLC cells [7, 8]. Basically, the full-length EML4 protein consists of the N-terminal coiled-coil trimerization domain and the C-terminal of the ALK protein, which contains the kinase domain. These domains are able to form a dimer without ligand binding, leading to activation of the ALK protein [9, 10]. And EML4-ALK fusion seems to be unique to NSCLC [11, 12]. Sequentially, a transgenic mouse model specifically expressing EML4-ALK in lung alveolar epithelial cells has been established to confirm its potent oncogenic activity [13]. Upon transcription of EML4-ALK, Mitogen-Activated Protein Kinase (MAPK), Janus Kinase with Signal Transducer And Activator Of Transcription (JAK-STAT) and Phosphoinositide-3-Kinase with VAkt Murine Thymoma Viral Oncogene Homolog (PI3K-AKT) are constitutively activated. There is evidence that these signaling pathways enhance proliferation, survival and angiogenesis in cancer cells [9, 14].

Besides, the corresponding targeted small molecule tyrosine kinase inhibitors (TKIs) have also led to unprecedented survival benefits in NSCLC patients with ALK rearrangement [15, 16]. In 2011, the first-generation ALK-TKI, crizotinib was approved for treatment of advanced ALK-positive NSCLC, which is a small molecule ATP-competitive ALK inhibitor [17]. In order to overcome crizotinib resistance, the second-generation ALK-TKIs, including ceritinib, alectinib and brigatinib were developed [18]. ALK TKIs have the potential to inhibit ALK phosphorylation and downstream signalling, leading to cell cycle arrest in the G1-S phase and apoptosis of cancer cells [19].

Typically, VTE occurs as a result of blood stasis, endothelial or vessel wall injury, and hypercoagulability. While cancer patients’ blood stasis and endothelial injury could be shared with non-cancer patients, the hypercoagulability driven by malignancy-specific pathways is probably unique to cancer. Previous studies have reported various mechanisms of hypercoagulation in malignancy, including indirect regulatory mechanisms such as expressing proteins by tumor cells that could alter circulating cells [20–23], and direct regulatory mechanisms such as expressing procoagulant proteins (TF, podoplanin (PDPN)) which directly activate the coagulation cascade/platelets [24–27].

Whether EML4-ALK fusion protein in NSCLC cells also enhances venous thrombogenicity through the mechanisms mentioned above is still unclear. We performed the following experiments to address this issue further and explore possible new targets for anticoagulation therapy in EML4-ALK-rearranged NSCLC patients.

## Materials and methods

### Cells and reagents

The ALK-positive human NSCLC cell lines H3122 and H2228 (purchased from Jiangsu KeyGEN BioTECH Co., Ltd, Jiangsu, China) and ALK-negative human NSCLC cell lines H1299 and A549 (purchased from FuHeng Biology, Shanghai, China) were maintained under a humidified atmosphere of 5% CO2 at 37 °C in RPMI medium 1640 supplemented with 10% fetal bovine serum, penicillin (100 U/ml) and streptomycin (0.1 mg/ml). Alectinib (catalog #S2762, Selleckchem, Houston, USA), SCH772984 (catalog #S7101, Selleckchem, Houston, USA), and T-5224 (catalog #GC16165, GlpBio, Montclair, USA) were each dissolved in dimethyl sulfoxide (DMSO). All reagents were stored at -20 °C or -80 °C.

### Western blot analysis

H3122 and H2228 cells were immediately lysed before use in RIPA Lysis Buffer (Solarbio, Beijing, China) supplied with protease inhibitor cocktails (KeyGEN BioTECH, Jiangsu, China) and phosphatase inhibitor cocktail (KeyGEN BioTECH, Jiangsu, China). Quantification of protein was determined by a Bicinchoninic Acid Assay (Thermo Fisher Scientific, Waltham, USA). Equal amounts of protein were subjected to 10% SDS PAGE and then transferred onto PVDF membranes. After incubated in blocking solution (NCM Biotech, Suzhou, China), specific antibodies for phospho-ALK (catalog #3341S, rabbit, 1:500, Cell Signaling, Beverly, USA), ALK (catalog #3633T, rabbit, 1:1000, Cell Signaling, Beverly, USA), TF (catalog #228,968, rabbit, 1:1000, Abcam, Cambridge, USA), phospho-ERK1/2 (catalog #4370, rabbit, 1:1000, Cell Signaling, Beverly, USA), ERK1/2 (catalog #9102, rabbit, 1:1000, Cell Signaling, Beverly, USA), c-FOS (catalog #2250, rabbit, 1:500, Cell Signaling, Beverly, USA) and GAPDH (catalog #5174S, rabbit, 1:3000, Cell Signaling, Beverly, USA) were used to incubate blots overnight at 4℃, then followed by incubating in HRP-conjugated goat anti-rabbit antibody for 1 h at room temperature and detecting the bands with the ECL system (Millipore Sigma).

### High-throughput sequencing

High-throughput sequencing was performed to detect different mRNA expressions between H3122 treated with Alectinib (100nmol/L, 6 h) and DMSO. CapitalBio Technology (Beijing, China) used an Illumina NovaSeq 6000 sequencer with a pair end 150-bp reading length (Illumina, San Diego, USA) to sequence mRNA and performed the final data analysis. The screening criteria for differential genes were: |log2FC|>=1 and p-Value < = 0.05. Cluster software was used to depict a heatmap for gene clustering.

### RNA isolation, reverse transcription, and quantitative RT-PCR

Total RNA extraction was performed by decomposing cells in TRIzol (Tiangen Biotech, Beijing, China). A reverse Transcription Kit (Takara Bio, Beijing, China) with random primers was used to synthesize cDNA. Quantitative real-time PCR (RT-PCR) analysis was performed by SYBR Green I master (Roche, Basel, Switzerland) and LightCycler480 II (Roche, Basel, Switzerland). The primer sequences were listed as follows: Human F3: forward primer 5’-GGCGCTTCAGGCACTACAAA-3’ and reverse primer 5’- CGTGCCAAGTACGTCTGCTT-3’; Human cfos: forward primer 5’- CACTCCAAGCGGAGACAGAC-3’ and reverse primer 5’- AGGTCATCAGGGATCTTGCAG-3’; Human HPRT-1: forward primer 5’-CCTGGCGTCGTGATTAGTGAT-3’ and reverse primer 5’- AGACGTTCAGTCCTGTCCATAA-3’. The results were calculated using the 2^−ΔΔCT^ method, and HPRT-1 served as a reference gene. Each sample was assayed at least in triplicate.

### Immunohistochemical analysis of human specimens

Human NSCLC tissues were obtained from patients at the Beijing Chao-Yang Hospital, Capital Medical University, with the approval of the institutional review board. For all patients involved in this study, amplification refractory mutation system polymerase chain reaction (ARMS-PCR) and Ventana immunohistochemistry were performed to detect ALK rearrangement. And the patients with ALK-rearrangement were confirmed by fluorescence in situ hybridization (FISH). The patients’ demographic and clinical data are presented in Table 1. The cancer tissues were formalin-fixed and paraffin-embedded. Anti-Tissue Factor antibody (catalog #228,968, 1:500; Abcam, Cambridge, USA) was used to detect TF. Images were obtained using a Leica TCS SP5 (Wetzlar, Hesse, Germany), and immunohistochemical staining of TF was determined using Images-Pro Plus version 6.0 software (IPP6) to assess the integrated optical density (IOD) and mean density of the immunohistochemical staining section. Finally, the mean IOD and density of cancer tissue immunohistochemical staining from five randomly selected fields (magnification, ×400) were recorded and analyzed.

**Table 1:** Information of patients for immunohistochemistry

Characters	Groups	ALK positive NSCLC Fraction	ALK negative NSCLC Fraction
Age(y)	< 60	6/10	2/17
	≥ 60	4/10	15/17
Sex	Male	4/10	5/17
	Female	6/10	12/17
Tumor histology	Adenocarcinoma	10/10	17/17
	Non-adenocarcinoma	0/10	0/17
Stage	I-IIIA	10/10	17/17
	IIIB-IV	0/10	0/17

### Flow cytometry and immunofluorescence

Cells (H3122, H2228, H1299 and A549) were surface-stained with BD Pharmingen™ PE Mouse anti-human CD142 (catalog #550,312, BD Bioscience, Franklin Lakes, USA) for 20 min at 4 °C and washed with PBS. Then cells were resuspended in a cell buffer solution at a concentration of 1 × 10^6^/mL for flow cytometric analysis (FACSCanto II; BD Bioscience) and analyzed using BD FCSDiva Software and FCS Express 5 software (De Novo Software, Los Angeles, USA).

H3122 cells in 6 wells were fixed in 4% paraformaldehyde and incubated with immunofluorescence mAbs against TF (Affinity Biosciences, Cincinnati, USA) overnight at 4 ℃, then followed by incubating in Alexa Fluor® 488-labeled goat anti-rabbit IgG for 1 h at room temperature and DAPI solution for 7 min. Images were obtained using a Leica TCS SP5 (Wetzlar, Hesse, Germany).

### Plasma clotting assay

Plasma clotting assay was performed to measure the plasma clotting time induced by lung cancer cells supernatant. Lung cancer cells (1 × 10^6^) were cultured in a complete medium for 24 h. Then, the cell culture supernatant was collected in an Eppendorf and centrifuged (3000 rpm for 10 min at 4℃) to remove cell debris. 200 ul of cell culture supernatant and 200 ul of 25 mM/L CaCl_2_ were added to 200 ul of citrated human plasma (healthy volunteers) at 37℃ to initiate the plasma clotting process. Samples contained both TF, pro-coagulants-bearing extracellular vesicles and tumor-secreted soluble pro-coagulants. Clotting time was recorded visually by noting when the liquid formed a semisolid gel that did not flow during tube turning over [24].

### Mouse model

The animal experiments were approved by the Institutional Animal Care and Use Committee of the Beijing Chaoyang Hospital, the Capital Medical University. Four-week old male athymic nude mice (BALB/c Nude, Vital River Laboratory Animal Technology, Beijing, China) were used to prepare the xenograft model. Human lung cancer cells (H2228, H1299) at a concentration of 1 × 10^7^ cells in 200uL of suspension were injected using 1 ml syringe into the subcutaneous tissue on the backs of mice (n = 6). And the control group was injected with PBS (n = 6). The tumor size was measured weekly until the volume was 200mm^3^ (about 3–4 weeks). When the tumor volume reached 200mm^3^, IVC model was developed. The inferior vena cava (IVC) ligation model was performed as described in the previous study [28, 29]. IVC was separated from the aorta after the laparotomy, and was ligated distal to the renal veins by using a 6 − 0 silk suture. To induce thrombus formation within the IVC, the tributaries surrounding the IVC were also ligated to create a total stasis environment. And 48 h later, clots were collected from the IVC and weighed. The clot weight was adjusted by body weight (clot weight/body weight).

Blood collected from the orbital sinus of mice was pipetted into sodium citrate tubes and placed in a centrifuge at 3000 rpm for 10 min at 4℃ to separate the plasma. Blood samples were not performed to remove pro-coagulants-bearing extracellular vesicles and tumor-secreted soluble pro-coagulants.

The experiments above were repeated three times.

### TF activity assay

TF procoagulant activity was assessed using a Tissue Factor Chromogenic Activity Kit (catalog #CT1002b, ASSAYPRO, Missouri, USA) as the manufacturer’s protocol.

### Statistics

Summary statistics are presented as mean ± SEM (standard error of the mean). A Student t-test and one-way ANOVA followed by LSD or Tamhane test were performed to analyze statistical comparisons between groups. IBM SPSS Statistics 22 software (IBM SPSS Statistics, IBM, Chicago, IL, USA) was used for data analysis and Graph Pad Prism 7 software (San Diego, CA, United States) was used for graphing. Statistical significance was assessed at the *P* <0.05 level.

## Results

### Targeting phosphorylated EML4-ALK fusion protein activity inhibits coagulation factor TF expression

**Fig. 1:**
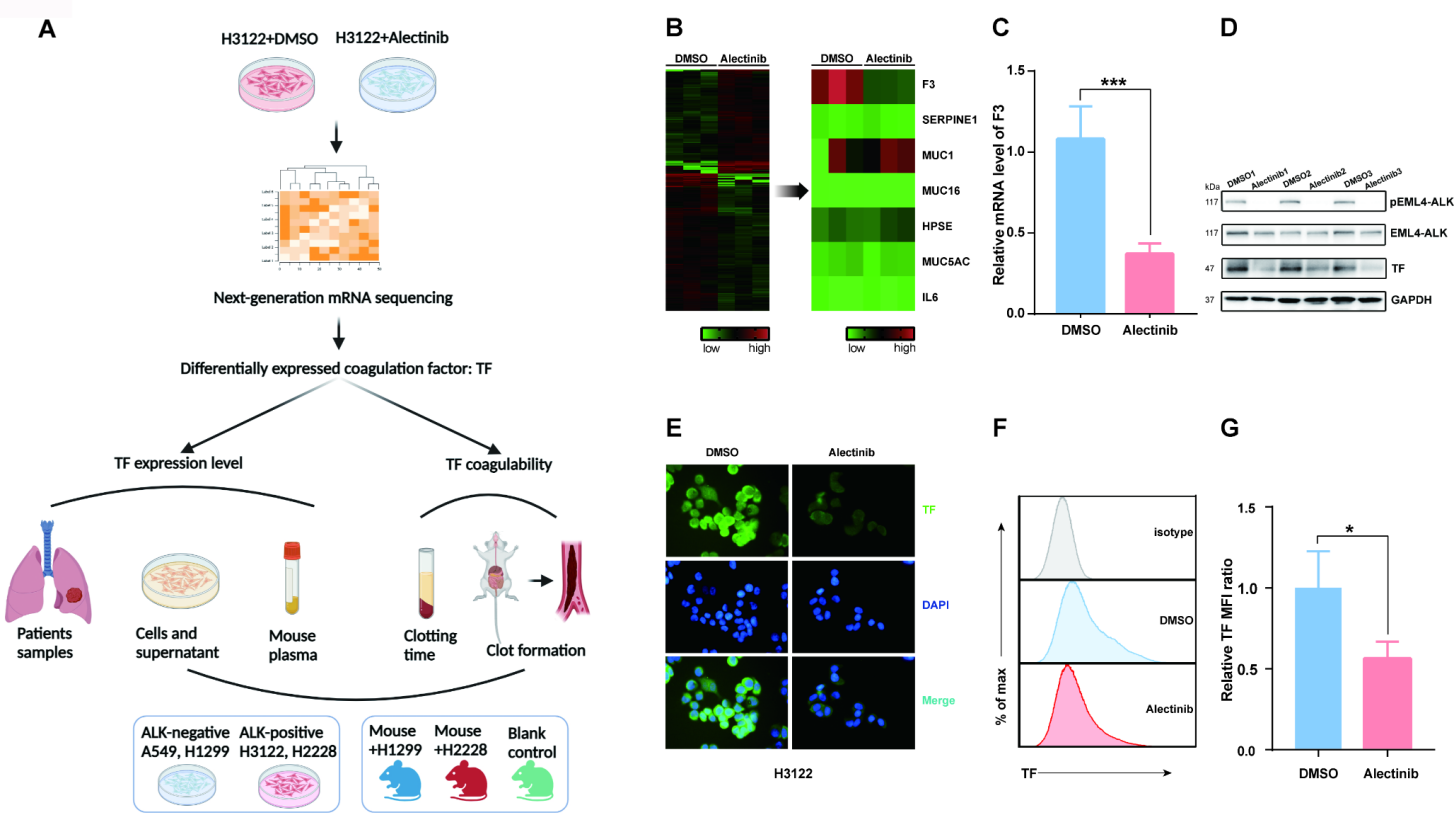
Targeting phosphorylated EML4-ALK fusion protein activity inhibits coagulation factor TF expression. (**A**) Schematic figure of the study. (**B**) Next-generation mRNA sequencing showed Treatment of ALK rearrangement human lung cancer cell line H3122 with Alectinib (100nmol/L, 6 h) substantially change the mRNA expression profile compared with DMSO-treated (vehicle-treated) H3122 cells; Among the genes involved in tumor-related coagulation, F3 gene was significantly downregulated in Alectinib-treated group (logFC =-1.54, P < 0.001). (**C**) mRNA expression of F3 gene in H3122 cells treated with Alectinib /DMSO was quantified by quantitative RT-PCR (n = 5). (**D**) Western blot of TF in H3122 cells treated with Alectinib /DMSO (n = 3). (**E**) H3122 cells stained with immunofluorescent anti-TF mAb to identify the localization of TF. (**F**) Flow cytometric analysis of TF expression at H3122 cell surface. (**G**) The mean fluorescence intensity (MFI) of anti-TF antibody in flow cytometry significantly decreased in Alectinib-treated group (P = 0.038) (n = 3). Data are presented as means ± SEM. *p < 0.05, ***p < 0.001, determined by Student t test

The schematic figure of the study is shown in Fig. 1.A. Treating H3122 cells, a validated EML4-ALK-positive NSCLC cell line, with Alectinib (100nmol/L, 6 h) [30]. Alectinib is a highly selective ALK inhibitor that showed strong antitumor activity against cancer cells which could achieve target inhibition of EML4-ALK fusion protein autophosphorylation, and substantially change the mRNA expression profile compared with DMSO in high-throughput mRNA sequencing analysis (Fig. 1.B). Besides, among the genes involved in aforementioned tumor-related coagulation [23, 31, 32], F3 gene was significantly downregulated in Alectinib-treated group (logFC =-1.54, P < 0.001) (Fig. 1.B). Subsequentially, using RT-PCR and immunoblotting, we confirmed that F3 mRNA expression and TF protein were much lower in H3122 cells after being treated with Alectinib (Fig. 1.C and Fig. 1.D).

To further investigate the expression and localization of TF in H3122 cells, immunofluorescence and flow cytometry were performed, and the results indicated that TF was mainly presented in the cell membrane (Fig. 1.E), and the mean fluorescence intensity (MFI) of anti-TF antibody in H3122 cells was also significantly decreased in Alectinib-treated group (P = 0.038) (Fig. 1.F and Fig. 1.G).

### ALK-positive lung cancer cell with high TF expression enhances clot formation

**Fig. 2:**
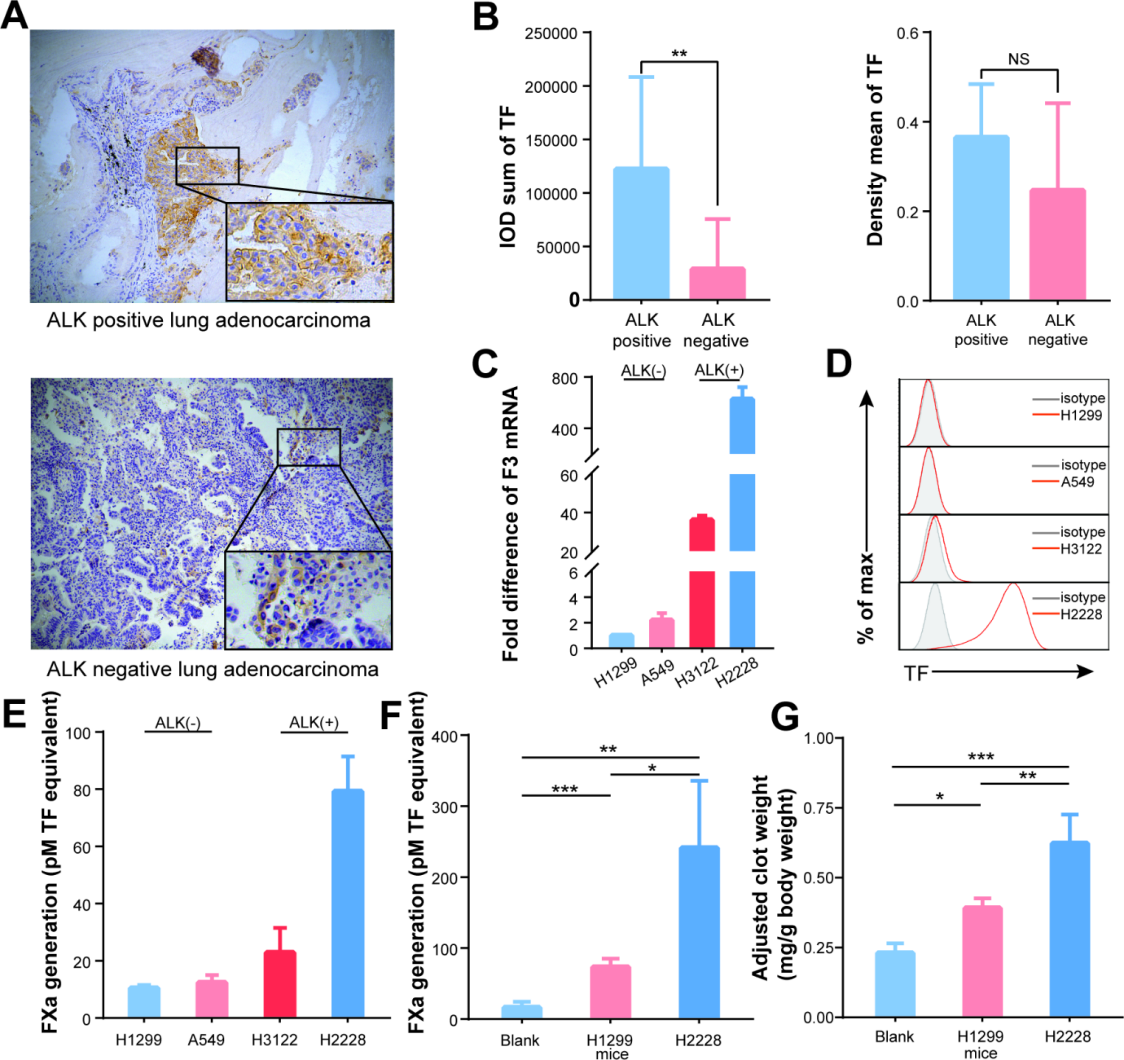
ALK-positive lung cancer cell with high TF expression enhances clot formation. (**A**) Primary lung cancer tissues obtained from ALK positive and ALK negative lung cancer patients. Tissue sections were stained immunohistochemically with anti-TF mAb. ALK positive NSCLC cancer cells express TF protein mainly on the cell membrane (upper panel); TF protein were negatively expressed in most ALK negative NSCLC cancer cells (lower panel). (**B**) Quantifying the immunochemical staining results with IPP6. ALK positive NSCLC tissues presented higher IOD value (left panel, P = 0.001). There was no significant difference in mean optical density value (right panel, P = 0.096). (**C**) The mRNA expression of F3 gene in ALK positive NSCLC lung cancer cell lines (H3122 and H2228) and negative NSCLC lung cancer cell lines (H1299 and A549) was quantified by quantitative RT-PCR (n = 3). (**D**) The expression of TF protein in ALK positive and negative NSCLC cell lines was quantified by Flow cytometry analysis. (**E**) TF activity of culture supernatant obtained from cell line cultures. (**F**) TF activity in plasma of mice bearing NSCLC cell lines and blank controls. (**G**) Clot weight in mice bearing different NSCLC cells and blank control after adjusted body weight (each n = 3 to 6). Data are presented as means ± SEM. *p < 0.05, **p < 0.01, NS, no significance, determined by Student t test or one-way ANOVA followed by Bonferroni test

Given the effect of Alectinib on regulating TF expression, we further sought to explore the expression of TF in ALK-positive and ALK-negative NSCLC patients and cell lines. First, we determined the expression of TF in NSCLC patients, and the characteristics of the patients were listed in Table 1. Ten ALK-positive NSCLC patients and 17 ALK-negative NSCLC patients were included, and the immunohistochemical staining results showed that TF was predominantly expressed at the tumor site in ALK-positive NSCLC (Fig. 2.A). Furthermore, quantifying the immunochemical staining results with IPP6, we also observed that ALK-positive NSCLC presented a higher IOD value than ALK-negative NSCLC (Fig. 2.B, P = 0.001), which was proportional to the total amount of expression of TF. However, there was no significant difference in the mean optical density value (Fig. 2.B, P = 0.096), which reflects the intensity of TF protein.

Next, TF expression was evaluated in ALK-positive NSCLC lung cancer cell lines (H3122 and H2228) and ALK-negative NSCLC lung cancer cell lines (H1299 and A549). RT-PCR showed that ALK-positive NSCLC cell lines exhibited a higher level of F3 mRNA expression, especially the H2228 cell line, whose expression level was about 625-fold higher than that of the H1299 cell line (Fig. 2.C). Consistent with the RT-PCR results, flow cytometry also confirmed that the H2228 cell line expressed the highest TF protein (Fig. 2.D). Similarly, the TF activity of culture supernatant obtained from H2228 cultures was the highest (79.2 ± 5.0pM), followed by H3122 (22.9 ± 3.5pM), A549 (12.4 ± 1.1pM), and H1299 (10.5 ± 0.4pM) (Fig. 2.E). In addition, we also performed a plasma clotting assay using the culture supernatant of each NSCLC cell line to assess their prothrombotic potent, as TF in the culture supernatant can activate coagulation factors in platelet-poor plasma and finally lead to insoluble fibrin formation. Consistent with the level of TF expression in NSCLC cell lines, the plasma clotting time of culture supernatant obtained from H2228 cultures was the shortest (45.3 ± 1.9s), followed by H3122 (121.7 ± 9.6s), A549 (602 ± 2s) and H1299 (621.7 ± 26.3s) (Supplementary Fig. 1.A).

Next, the expression of tumor-derived TF in circulation was further examined. As soon as the xenograft model tumor volume reached 200 mm^3^, plasma was collected for ELISA detection of tumor-derived TF concentration. Meanwhile, TF activity (tumor-derived and mice-derived) in the plasma of mice bearing H2228 is highest (H2228 vs. H1299, P = 0.021; H2228 vs. blank control, P = 0.006), followed by H1299 and blank control (H1299 vs. blank control, P < 0.001) (Fig. 2.F). After that, the IVC ligation model was also developed on blank mice and mice bearing H1299 and H2228 tumors. The results indicated that clot weight increased in mice with NSCLC when compared to blank mice after adjusting body weight (clot weight/body weight) (blank control vs. H1299, P = 0.025; blank control vs. H2228, P < 0.001). Also, consistent with the level of TF concentration and activity of NSCLC cell lines, the clot weight in mice bearing H2228 tumors was significantly higher than those bearing H1299 tumors (H2228 vs. H1299, P = 0.005) (Fig. 2G).

### EML4-ALK fusion protein regulates TF expression through the pERK1/2-AP-1 pathway in NSCLC cells

The promoter region of the F3 gene contains multiple elements for diverse transcription factors binding [33], and phosphorylated EML4-ALK fusion protein in NSCLC cells could activate multiple downstream pathways [15]. We further analyzed the results of high-throughput mRNA sequencing for transcription factors that may regulate F3 gene expression and KEGG pathways in which EML4-ALK fusion protein is involved. The results implied that cfos mRNA expression, whose protein product was one of the subunits comprising F3 gene transcription factor AP-1 [34–36], was also down-regulated along with the F3 gene. Besides, the upper regulatory pathway of AP-1 overlapped with the downstream pathway of EML4-ALK at the node of ERK1/2 [37, 38].

**Fig. 3:**
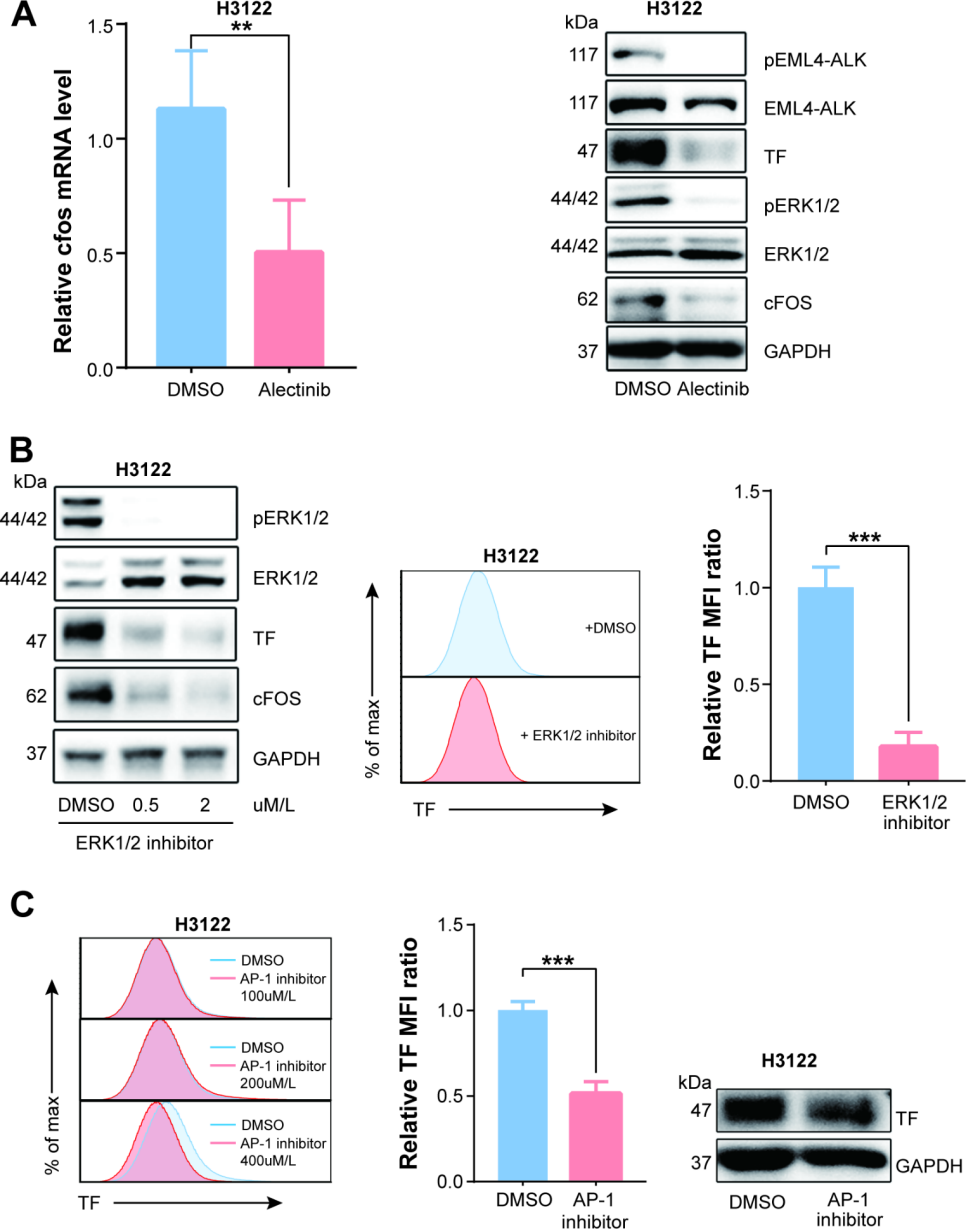
EML4-ALK fusion protein regulates TF expression through pERK1/2/AP-1 pathway in H3122 cells. (**A1**) The mRNA expression of cfos gene in H3122 cells treated with Alectinib and DMSO was quantified by quantitative RT-PCR (n = 6). (**A2**) The expression of pERK1/2 and cfos protein in H3122 cells treated with Alectinib and DMSO was quantified by Western Blot (n = 3). (**B1**-**3**) Pretreating H3122 cells with ERK1/2 inhibitor (SCH772984) downregulated ERK1/2 phosphorylation, cfos and TF protein expression compared with DMSO control which identified by Western Blot and flow cytometry (n = 3). (**C1**-**3**) Pretreating H3122 cells with AP-1 inhibitor (T-5224) also attenuated TF expression compared with DMSO control which identified by Western Blot and flow cytometry (n = 3). Data are presented as means ± SEM. **p < 0.01, ***p < 0.001, determined by Student t test

The results verified that cfos gene expression was downregulated with F3 in Alectinib-treated H3122 cells through RT-PCR (Fig. 3.A, left panel). Further, pERK1/2 and cfos protein expression were also decreased in Alectinib-treated H3122 using Western Blot (Fig. 3.A, right panel).

Next, we determined TF and cfos expression in H3122 cells after SCH772984 treatment. SCH772984 is a selective inhibitor of ERK1/2, which adopts a unique kinase binding mode in ERK1/2 [39]. Pretreated H3122 cells with SCH772984 (2 μm/L, 24 h) to downregulate ERK1/2 phosphorylation, and compared cfos and TF protein expression with DMSO control. The results showed that cfos and TF proteins were downregulated when ERK1/2 phosphorylation was inhibited (Fig. 3B). Also, in line with the ERK1/2 inhibitor, pretreated H3122 cells with T-5224 (400nmol/L, 24 h), a small molecule selective inhibitor that selectively inhibits c-Fos/AP-1 binding to DNA [40], TF expression was downregulated compared to DMSO control (Fig. 3C).

**Fig. 4:**
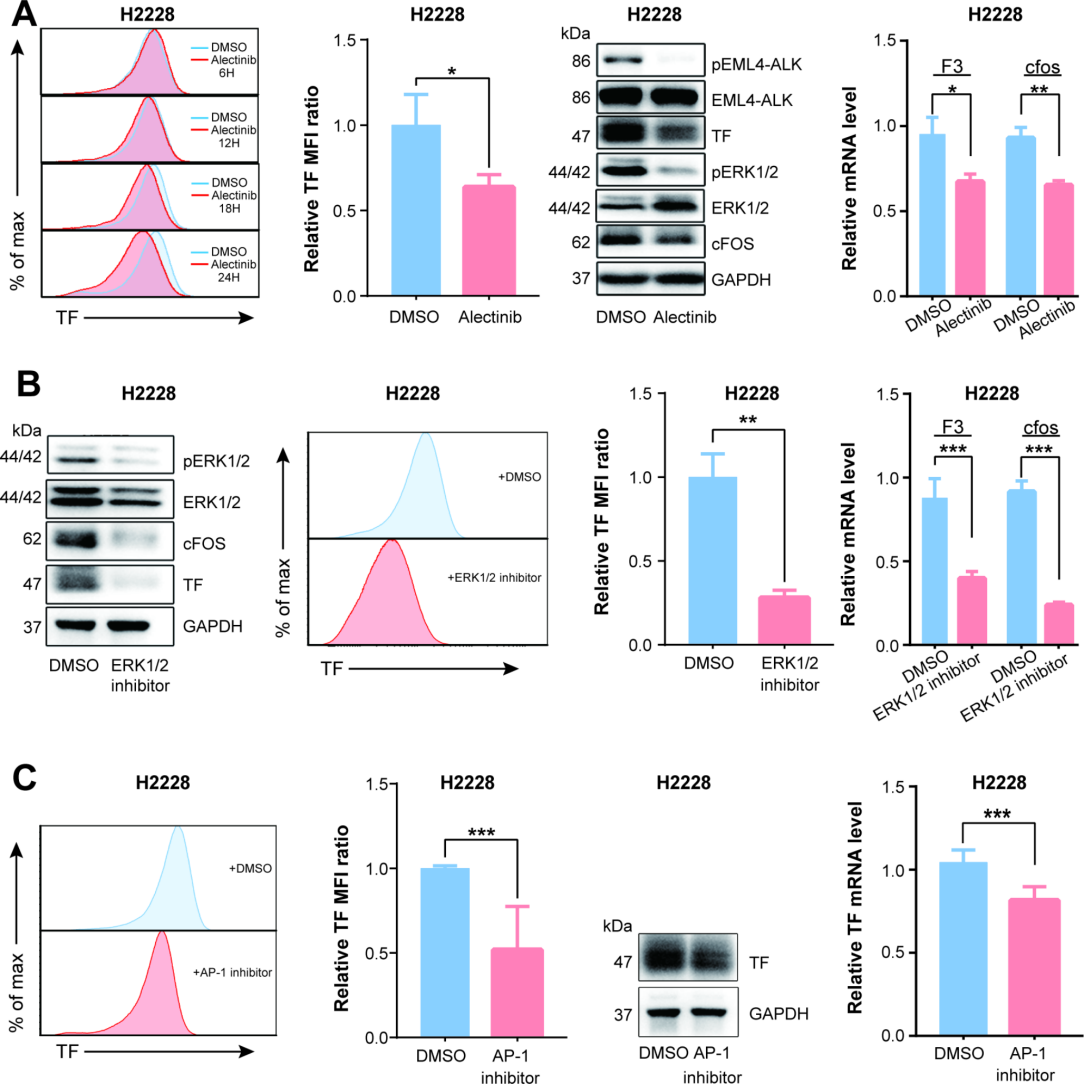
EML4-ALK fusion protein regulates TF expression through pERK1/2/AP-1 pathway in H2228 cells. (**A1**-**2**) Pretreating H2228 with the same concentration of Alectinib in H3122. Flow cytometry analysis showed that TF expression was significantly down regulated at 18 h and even more obvious at 24 h (n = 3). (**A3**) Western Blot was observed decreased pERK1/2 and cFOS along with TF down regulation (n = 3). (**A4**) F3 gene and cFOS mRNA expression were similarly reduced compared with DMSO control which quantified by quantitative RT-PCR (n = 4). (**B1**-**4**) Pretreating H2228 cells with ERK1/2 inhibitor (SCH772984, 2 μm/L)) downregulated ERK1/2 phosphorylation, cfos and TF protein expression compared with DMSO control both at RNA (n = 6) and protein level (n = 3). (**C1**-**4**) Pretreating H2228 cells with AP-1 inhibitor (T-5224, 400 μm/L) also attenuated TF expression compared with DMSO control both at RNA (n = 6) and protein level (n = 3). Data are presented as means ± SEM. **p < 0.01, ***p < 0.001, determined by Student t test

After evaluating the mechanism of EML4-ALK regulating TF through the ERK1/2 /AP-1 axis in H3122 cells, we further verified these observations in H2228 cells. When H2228 was pretreated with the same concentration of Alectinib, flow cytometry analysis showed that TF expression was significantly down-regulated at 18 h and even more evident at 24 h (Fig. 4.A, left two panels). Western Blot results indicated that pERK1/2 and cFOS proteins were decreased with TF downregulation (Fig. 4A, middle panel). Also, mRNA expression of cFOS and F3 genes was both reduced after Alectinib treatment (Fig. 4A, right panel). In addition, ERK1/2 and AP-1 inhibitors also reduced the expression of TF mRNA and protein in H2228 cells (Fig. 4B and C).

## Discussion

**Fig. 5:**
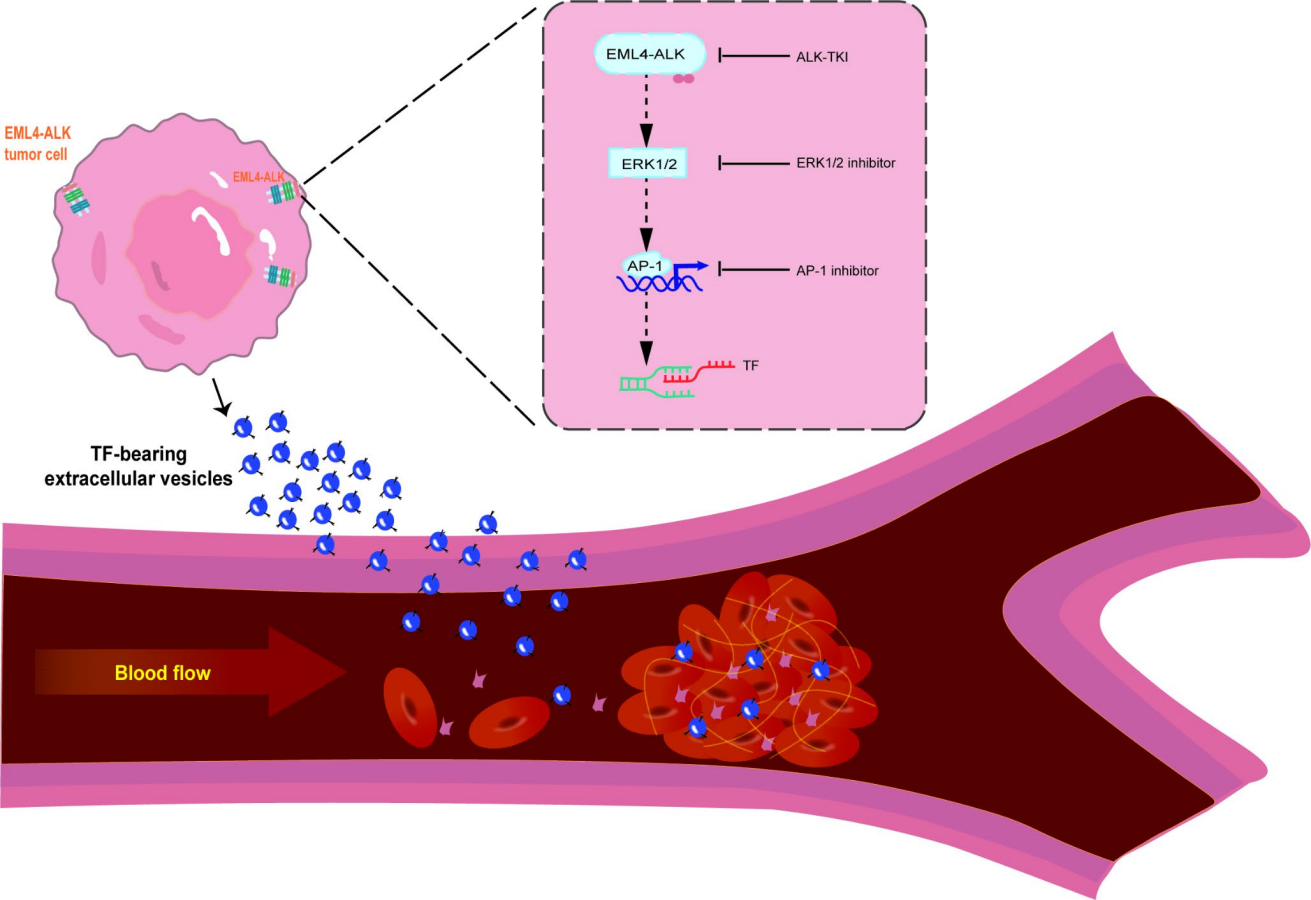
Summary scheme graphic showing an association between EML4-ALK fusion protein mediated cellular pathway and thrombosis. Phosphorylated EML4-ALK fusion protein activated downstream the ERK1/2 pathway, inducing cFOS expression which was one of subunit of transcription factor AP-1. Thus, in turn result in upregulation of TF expression. Consequentially, tumor-derived TF in circulation triggered coagulation cascade

In recent decades, an increasing number of studies have found that ALK-rearranged NSCLC patients have a higher risk of VTE occurrence [41, 42]. Besides, a large proportion of these VTE events are developed in newly diagnosed NSCLC patients, supporting the underlying cancer-specific biology as a causal factor. According to previous clinical studies, the association between EML4-ALK rearrangement and VTE occurrence is verified. Thus, in this study, we firstly demonstrated that TF might be the key regulator of thrombus formation in EML4-ALK rearranged NSCLC in both in vitro and vivo experiments. Meanwhile, we also observed in vitro that EML4-ALK fusion protein in NSCLC cells enhanced venous thrombogenicity via pERK1/2 mediating AP-1-TF signaling. Consequentially, tumor-derived TF in the circulation triggered a coagulation cascade (Fig. 5).

This research indicated that the EML4-ALK-pERK1/2-AP-1-TF axis might be a potential mechanism of VTE in ALK-rearranged NSCLC patients. The nodes of this axis also play essential roles in cancer initiation and progression [43–45], and both of them are therapeutically targetable proteins, which implies an important translational potential for the treatment of cancer-associated VTE.

### Cancer-specific mechanisms of VTE in EML4-ALK fusion NSCLC cells

VTE occurrence in malignant patients could be due to multiple factors, including patient characteristics (like age, sex, and co-morbidities), cancer treatment (like chemotherapy, radiotherapy, and anti-vascular therapy), and cancer itself. Recent studies have discovered various cancer-specific mechanisms of VTE in brain, pancreatic, and colon cancer. These mainly included cancer cells expressing proteins that could alter the number of platelets and leukocytes in circulation (such as IL6, MUC1, MUC16, and MUC5AC) [46] and the expression of procoagulant proteins such as TF and PDPN, which could directly activate the coagulation cascade and upregulation of antifibrinolytic/anticoagulation proteins (PAI1 and HPSE) [47–50].

TF protein is a 47 kDa transmembrane protein that is highly expressed in many human cancers, including glioma, pancreatic, head, neck, lung, cervical, and prostate cancers, as well as leukemia [51]. Besides, an alternative spliced (as) form of TF that lacks the transmembrane domain can be released from cells. But studies about procoagulant activity of asTF are inconsistent [52–54]. So, the researches mostly focus on the full-length TF (TF). Studies have shown that tumor growth and angiogenesis are mediated by TF expression, and that TF expression correlates directly with oncogenic status, while circulating TF-positive extracellular vesicle level correlates with oncogenic status as well [55]. And tumor TF expression level is proven to influence cancer prognosis [55]. TF initiates the extrinsic coagulation cascade and can be released into circulation through cell-derived extracellular vesicles [56]. When TF-positive extracellular vesicles shed from cancer cells are associated with coagulation factor VII (FVII), this can trigger the blood coagulation cascade, leading to cancer-associated VTE [55]. There is no evidence that cancer cells-derived TF is activated in the circulation.

In the current research, as cohort studies indicated a heightened risk of VTE occurrence in patients with ALK-rearranged NSCLC [4, 6], we targeted inhibition of EML4-ALK fusion protein in NSCLC cells and observed that TF expression was significantly decreased. And this finding is similar to previous studies that increased cell surface expression of TF was associated with higher procoagulant activity in malignancies and higher circulating levels in vivo [57, 58], which was also shown in breast and ovarian cancer patients [59, 60]. Thus, inhibiting EML4-ALK fusion protein in cancer cells might decrease coagulability. Previous studies detected tumor-derived TF-positive extracellular vesicles (micropaticles or microvesicles) in the plasma of human pancreatic/colorectal tumor cells xenografted mice and examined venous thrombogenicity in a mouse model [61–64]. In addition, we demonstrated the function of TF by testing the activity of TF in human ALK-positive/ALK-negative NSCLC cell lines and also in mice bearing tumors derived from the corresponding cell lines. Furthermore, EML4-ALK fusion NSCLC cell lines with higher TF expression showed shorter clotting time in their culture supernatant involving plasma clotting assay. As a result, the plasma clotting assay might be influenced by samples containing TF-positive extracellular vesicles and tumor-secreted soluble procoagulants. And further experiments should be performed to verify this result.

Also, several studies within different cancer types indicated that increased tumor-derived TF-positive microvesicles upregulated the VTE incidence and venous clot weight in IVC stenosis or ligation mouse models [28, 29, 63]. In addition, Zwicker et al. proved that tumor-derived TF-positive microvesicles are associated with VTE in cancer patients [65]. Our findings are consistent with these results. Additionally, the present study has intensively considered oncogenic mutation and demonstrated that oncogenic mutation is a significant factor influencing cancer hypercoagulation.

We further identified TF expression in ALK-positive and ALK-negative NSCLC tissues and observed that ALK-positive NSCLC tissues exhibited higher TF expression. This finding was also indicated in a previous retrospective study, and the ALK-positive NSCLC patients in this cohort with higher TF expression showed a greater possibility of developing VTE compared to patients with EGFR mutation and both negative [66]. Collectively, the findings mentioned above suggest TF as the specific mechanism of EML4-ALK fusion in NSCLC-associated VTE.

### EML4-ALK-pERK1/2-AP-1-TF axis in EML4-ALK fusion NSCLC cells

The EML4-ALK fusion protein is the product of the EML4-ALK fusion gene caused by chromosome translocation. The EML4 locus located in the short arm of chromosome 2 is broken, inversed, and fused with the ALK locus located on the same chromosome in somatic cells [7]. Thus, the fusion protein is composed of the amino-terminal half of EML4 protein ligating to the intracellular region of the receptor-type protein tyrosine kinase ALK. This action leads to dimerization and autophosphorylation of the ALK kinase domain and thus abnormally activates downstream signaling pathways, such as PI3K/AKT, JAK/STAT3, and RAS/ERK [67, 68], finally acquiring tumor-formation activity [13].

The regulatory mechanism of TF expression has been reported in many cancer models containing brain, breast, and colorectal cancer [2, 24, 69–72]. These studies revealed that multiple signaling pathways, transcription factors, and microRNAs, such as the Raf-MEK-ERK signaling pathway, transcription factor AP-1, and nuclear factor κB (NF-κB) could regulate TF expression in cancer cells [70]. And the mammalian target of rapamycin (mTOR) kinase pathway was identified in human pancreatic neuroendocrine tumor cell lines [73].

In the current study, targeted inhibiting EML4-ALK fusion protein in NSCLC cells showed downstream suppression of pERK1/2 and reduction of cFOS, a subunit of AP-1, accompanied by downregulation of TF. It is similar to a previous study within breast cancer cells, in which ERK1/2 kinase activity was measured in nuclear extracts and shown to be upregulated in MDA-MB-231 breast cancer cells with higher expression of TF mRNA [70]. Also, both AP-1 and NF-κB are important transcription factors for TF expression. However, compared to NF-κb, MDA-MB-231 nuclear extracts contain a molar excess of AP-1 [70]. And in our study, targeting ERK1/2, we also observed the downregulation of the subunit of AP-1 and TF. Finally, after pretreating with an AP-1 inhibitor, there is a significant reduction of TF expression both in H3122 and H2228 cell lines. Nevertheless, the former study only investigated the regulation of TF expression in non-specific breast cancer, and in this study, we linked EML4-ALK oncogenic alteration, the activation of downstream signaling pathways, and the coagulation cascade. Overall, the current study implies that EML4-ALK fusion protein in NSCLC cells may regulate the expression of TF through the pERK1/2-AP-1 axis.

### The translational potential of the EML4-ALK-pERK1/2-AP-1-TF axis in the treatment of cancer-associated VTE

Thromboprophylaxis and anticoagulation therapy for cancer patients need to consider the risk of bleeding, the impact of the anti-tumor treatment process, and the additional sense of futility and burden caused by such treatment because it seems this action does not extend survival. Despite anticoagulation therapy, the incidence of recurrent pulmonary embolism (PE) remains relatively high [32, 74]. Hence, identifying patients at high risk of VTE is a current research focus. Notably, many studies suggested that high levels of TF expression were observed in different types of cancer, and the level of TF expression was associated with tumor progression and hypercoagulability [55]. Thus, TF expression might be a marker of cancer prognosis. However, little research on individualized anticoagulation therapy based on specific cancer types. This study aims to identify a non-anticoagulant target for VTE treatment in NSCLC with a specific oncogenic mutation.

This regulatory axis may be an appealing target for cancer-associated VTE in EML4-ALK fusion NSCLC patients. The nodes of this axis have essential roles in the occurrence and development of malignancy, and they all have corresponding specific targeted inhibitors. For example, small molecular ATP-competitive ALK inhibitors, which can effectively inhibit the autophosphorylation of ALK protein and suppress downstream signal activation [75, 76], have led to unprecedented survival benefits in EML4-ALK fusion NSCLC patients [77–79]. Although targeted ERK1/2 inhibitors are still in preclinical/clinical research [80, 81], inhibitors targeting upstream signaling protein MEK (RAS-RAF-MEK-ERK1/2 pathway) have been approved by FDA in the USA for the treatment of various solid tumors and have achieved excellent results in clinical [82–84]. Hrustanovic, Gorjan, and Tanizaki, J et al. [85, 86] found that EML4-ALK fusion NSCLC cells specifically depend on the MAPK pathway, and their sensitivity to MEK inhibitors is similar to that of KRAS or BRAF-positive lung adenocarcinoma cells. However, ALK inhibitors are not entirely effective, and single-drug treatment for long-term use can induce the reactivation of the downstream MAPK pathway and lead to drug resistance. Thus, a combined treatment strategy of initial ALK and MEK inhibitors is recommended to improve the survival rate of patients [87].

Now that the current data support the role of the axis in the pathogenesis of cancer-associated VTE in EML4-ALK fusion NSCLC cells, the node inhibitors may fulfill a dual purpose in patients with EML4-ALK fusion NSCLC. And the results suggest that clinicians should give careful consideration to providing thromboprophylaxis to NSCLC patients harboring EML4-ALK rearrangement. A non-anticoagulant target for VTE treatment might offer a new reference for thromboprophylaxis and anticoagulation therapy for cancer patients, and it might also provide more theoretical support for a combination treatment strategy of ALK and MEK inhibitors.

### Limitations

This study has the following limitations. First, the mouse model used in this study is a subcutaneous ectopic lung cancer model, which cannot fully represent the real pulmonary environment with rich blood circulation. This limitation can be addressed by building an orthotropic model in the future. However, due to differences in the immune response and tumour microenvironment between mice and humans, animal models may not fully recapitulate human disease physiology. Additionally, the complex heterogeneity and microenvironmental factors present in clinical settings may not be fully reflected in the results obtained from the limited cell line experiments. Thus, further in vivo experiments and clinical studies should be performed to confirm these results. And these results should be verified in more types of cell lines. Also, there is a lack of evaluation of TF concentration in peripheral blood of lung cancer patients in the current study, limited by the fact that the number of enrolled ALK-rearranged NSCLC patients is too small. A comprehensive analysis will be carried out after further expanding the sample size in the future.

## Conclusion

In summary, we have uncovered that EML4-ALK fusion protein in lung cancer cells enhances venous thrombogenicity through the pERK1/2-AP-1-tissue factor axis. This finding provides more theoretical support for a combination treatment strategy of ALK and MEK inhibitors and offers a new reference for thromboprophylaxis and anticoagulation therapy for cancer patients.

### Electronic supplementary material

Below is the link to the electronic supplementary material.

Supplementary Material 1
