# Supplementary material for: EML4-ALK fusion protein in Lung cancer cells enhances venous thrombogenicity through the pERK1/2-AP-1-tissue factor axis
Source: J Thromb Thrombolysis. 2023 Nov 8;57(1):67–81. doi: 10.1007/s11239-023-02916-5 (PMC10830642; doi:10.1007/s11239-023-02916-5)
Supplement: Supplementary file 1 — Supplementary Material 1 [file 11239_2023_2916_MOESM1_ESM.docx]

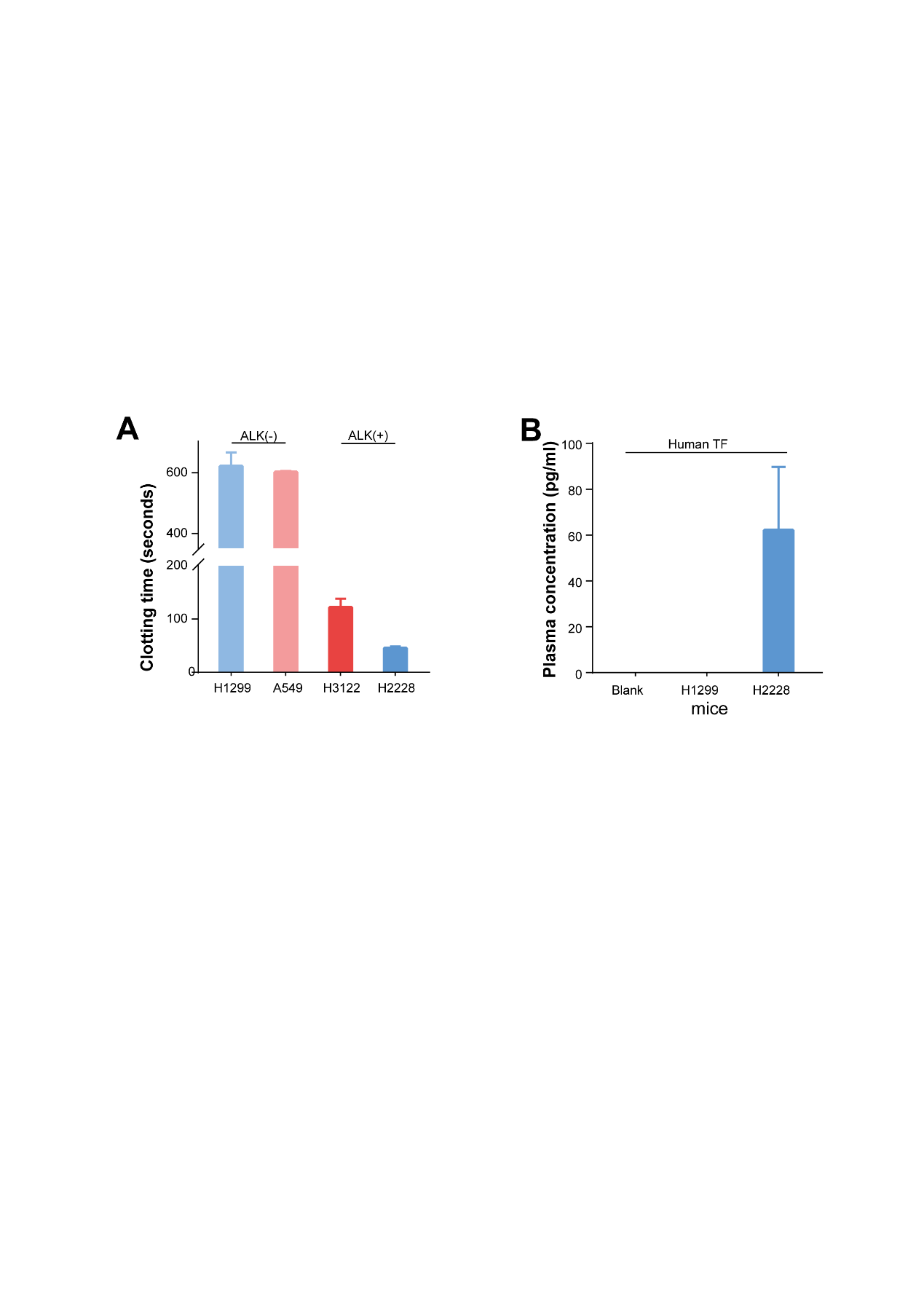


Supplement 1. (A) Plasma clotting time of culture supernatant obtained from cell line cultures. The plasma clotting time of culture supernatant obtained from H2228 cultures was 45.3±1.9s, followed by H3122 (121.7±9.6s), A549 (602±2s) and H1299 (621.7±26.3s).
